# Distance-Based Paper Device Combined with Headspace Extraction for Determination of Cyanide

**DOI:** 10.3390/s19102340

**Published:** 2019-05-21

**Authors:** Papichaya Khatha, Thanyarat Phutthaphongloet, Phenphitcha Timpa, Benjawan Ninwong, Kamolwich Income, Nalin Ratnarathorn, Wijitar Dungchai

**Affiliations:** 1 Organic Synthesis, Electrochemistry & Natural Product Research Unit, Department of Chemistry, Faculty of Science, King Mongkut’s University of Technology Thonburi, Prachautid Road, Thungkru, Bangkok 10140, Thailand; papichaya18@gmail.com (P.K.); thanyarat.phut@gmail.com (T.P.); f_phenphitcha@outlook.co.th (P.T.); ben-benone@hotmail.com (B.N.); kamolwich_in@hotmail.com (K.I.); Nalinratnarathorn@gmail.com (N.R.); 2 Applied Science & Engineering for Social Solution Unit, Faculty of Science, King Mongkut’s University of Technology Thonburi, Prachautid Road, Thungkru, Bangkok 10140, Thailand

**Keywords:** distance-based paper device, paper-based headspace extraction, cyanide

## Abstract

We report for the first time a distance-based paper device based on gold/silver core shell nanoparticles (Au@Ag NPs) for a simple, inexpensive, instrument-free, and portable determination of cyanide by the naked eye. Au@Ag NPs immobilized on a paper channel were etched by cyanide ions so that a yellow color band length of Au@Ag NPs is proportional to a decrease in the cyanide concentration. Quantification is achieved by measuring color length, thus eliminating the need to differentiate hues and intensities by the user, and the processing data of each imaging device. Moreover, the paper-based headspace extraction was combined with the distance-based paper device to improve the sensitivity. The enrichment factor was found to be 30-fold and the linearity was found in the range 0.05–1 mg L^−1^. The naked eye detection limit was 10 μg L^−1^ where the World Health Organization (WHO) have regulated the maximum level of cyanide in drinking water as 70 μg L^−1^. Our proposed device also showed no interference from common cations and anions presenting in seawater and waste water including thiocyanate, chloride. Finally, our device has been successfully applied to determine cyanide ions in seawater, drinking water, tap water and wastewater providing satisfactory precision and accuracy.

## 1. Introduction

Cyanide is a highly poisonous compound commonly used in industries such as gold mining, electroplating, burning of municipal waste and pesticides that can be released in the environment. The most toxic form of cyanide is free cyanide including cyanide ion and hydrogen cyanide (HCN). The cyanide remains in solution as ions at pH > 11, while the cyanide will exist as HCN at more acidic than pH 5 [1]. The World Health Organization (WHO) have regulated the maximum level of cyanide in drinking water as 70 µg L^−1^ and the U.S. Environmental Protection Agency (EPA) has established a maximum cyanide contamination level of 200 µg L^−1^ in ambient water [2]. In unpolluted air, the concentration of HCN is less than 0.2 parts of HCN per million of air volume [3]. Various methods for the determination of cyanide have been developed such as gas chromatography [4,5], electrochemistry [6,7], chemiluminescence [8], and capillary electrophoresis [9]. Although these methods provide a high sensitivity and selectivity, most of them require instruments or skilled technicians, and are consequently not suitable for field monitoring.

Recently, nanoparticle-based colorimetric sensors have attracted attention for their simple and high-throughput determination of cyanide [10,11]. The bimetallic nanoparticles for a cyanide colorimetric sensor have been also reported to improve visual resolution and provide a low detection limit of cyanide [12,13]. For example, Zeng et al. [12] found the detection limit of cyanide to 10.4 µg L^−1^ by using Au@Ag core shell nanoparticles. Li, et al. [13] reported Ag@Au core shell nanoparticles for the detection of cyanide with limit of detection (LOD) as 4.2 µg L^−1^ and the cyanide level can be semi-quantitatively detected by the naked eye. Unfortunately, these assays are limited by the need of a spectrometer for quantitative analysis as well as the large sample and nanoparticle reagent (mL) consumption. In an effort to conduct a quantitative analysis of cyanide using instrument-free, paper-based analytical devices, a smart phone and scanner assisted detection have the potential to be good alternatives over others due to their ease of use, low cost, less need for reagents and sample consumption, rapid analysis, and portability [14,15]. Although smart phones and scanners can distinguish changes in color intensity to quantify cyanide, there are some limitations. Smart phone-based detection needs a black cardboard box to eliminate the lighting effect [15]. Another assay cannot use to detect cyanide directly due to thiocyanate interference [14]. They need to quantify cyanide by using the difference between amount of thiocyanate and cyanide including thiocyanate. Furthermore, the recoveries of cyanide in spiked seawater was lower than 80% because they lack the interference study of some anions presenting in seawater such as chloride. To overcome their limitations, we developed distance-based paper device for the cyanide quantitative analysis by naked eye. The length of a yellow colored band of Au@Ag core shell nanoparticles from etching reaction increased linearly with cyanide concentration. Many researchers have applied distance-based paper device for the determination of glucose, nickel and glutathione [16], aerosol oxidative activity [17], chloride ion [18], lactoferrin [19], and heavy metals [16,20]. However, they had never been reported for the determination of cyanide. Moreover, paper-based headspace extraction has been developed to enhance the sensitivity and selectivity for sulfite [21], sulfur dioxide [22], volatile organic compounds [23], hydrogen sulfide [24], arsenic [25], and zinc [26] determination. Cyanide will exist as HCN at more acidic than pH 5 so paper-based headspace extraction can be applied for the highly sensitive and selective determination of cyanide.

In this work, a distance-based paper device coupled with headspace extraction was developed for the naked-eye detection of cyanide by using the etching reaction of Au@Ag core shell nanoparticles. When drop cyanide solution, a yellow color of Au@Ag core shell nanoparticles in a paper channel changes to colorless by cyanide etching reaction. Quantitative analysis can be achieved by measuring the length of the colorless band. Our proposed device shows high selectivity, low cost, simplicity, instrument-free and portability for the determination of cyanide by unskilled personnel. Finally, our device has been successfully applied to determine cyanide ions in real water samples providing satisfactory precision and accuracy.

## 2. Materials and Methods

### 2.1. Chemicals and Materials

Chloroauric acid trihydrate (HAuCl_4_∙3H_2_O), Silver nitrate (AgNO_3_), *L*-ascorbic acid (AA), Sodium hydroxide (NaOH), anion salts (Na_2_SO_4_, NaCl, NaNO_3_, Na_2_HPO_4_, NaHS∙H_2_O, NaF, NaBr, NaI and KSCN), cation salts (Al(SO_4_)_2_∙18H_2_O, CuSO_4_∙5H_2_O, ZnSO_4_∙7H_2_O, HgCl_2_, Pb(NO_3_)_2_, Fe(NO_3_)_3_, FeSO_4_, CaCO_3_, MgSO_4_∙7H_2_O, NiSO_4_∙6H_2_O, CrN_3_O_9_∙9H_2_O, CdSO_4_, CoSO_4_∙7H_2_O) were obtained from Merck, Germany. All chemicals used in experiment were analytical reagent (AR grade). 

### 2.2. Instrumentation

The absorption spectra were measured by UV-Visible spectrometer (Lambda 35, Perkin15 Elmer Instruments, Massachusetts City, MA, USA) at the wavelength 300–800 nm using quartz cuvettes (1.0 cm path length) to characterize gold nanoparticles (AuNPs), silver nanoparticles (AgNPs) and gold@silver core-shell nanoparticles (Au@Ag NPs). TEM images were performed using Transmission Electron Microscope (TEM, JEOL, JEM-2010) to analyze the nanoparticle sizes and shapes.

### 2.3. Synthesis of Gold, Silver and Gold@silver Core-Shell Nanoparticles

Gold nanoparticles (AuNPs) solution was prepared according to bio-green method and Turkevich method [27]. Briefly, 95 mL of 5 mM HAuCl_4_·3H_2_O in 0.01% (w/v) soluble commercial starch was freshly prepared and boiled for 30 min with stirring followed by adding 5 mL of 0.5% (v/v) tri-sodium citrate. Then, the solution was continually boiled for 15 min and cooled down to room temperature. The color change of AuNPs solution from pink to red wine was observed by naked eye. The shape and particle size distributions of the obtained AuNPs were confirmed with TEM.

Silver nanoparticles (AgNPs) solution was prepared by using silver nitrate (AgNO_3_) in the presence of starch solution as reducing agent. Briefly, the AgNO_3_ solution was added to starch solution drop by drop during at a flow rate of 2 mL s^−1^. Solution was stirred vigorously during this process at room temperature. For indicating the formation of the small silver nanoparticles, the color change was noticeable from colorless to dark yellow. Then, AgNPs solution was boiled for 2 h and cooled down to room temperature for 12 h. The shape and particle size distributions of the obtained AgNPs were confirmed with TEM.

For a preparation of Au@Ag NPs, 200 μL of 1000 mg L^−1^ AgNO_3_ was mixed with 300 μL of the prepared AuNPs solution acting as the core. Then, 200 μL of 500 mg L^−1^ ascorbic acid and 300 μL of 0.01 M NaOH were added to the mixture solution. After that, the solution turned into yellow immediately. Finally, the Au@Ag NPs products were characterized using TEM and UV-Visible spectrophotometry.

### 2.4. Device Fabrication 

Using CorelDraw to design the pattern of the device in the shape of a thermometer that consisted of a sample zone (diameter 7 mm) and a straight channel for detection zone with a width 2 mm and length of channel 40 mm. The width of the hydrophobic wax was 1 mm. Then, designed was printed onto Whatman No.1 filter paper using a wax printer (Xerox ColorQube 8870, Yokohama, Japan). The device was heated at 150 °C in an oven for 2 min and then cooled to room temperature. To prevent solution leakage through the device, tape was sealed with the back side of the device. For analysis, 7 μL of 2.19 × 10^−11^ mol L^−1^ Au@Ag NPs were spotted onto the detection channel and allowed to dry. After that, 50 µL of standard or sample solution was added onto the sample zone and flowed through the detection channel by capillary action.

### 2.5. Optimization of Detection Conditions on Distance–Based Paper Devices

The distance-based paper devices (dPADs) were optimized with various parameters. Firstly, 50 μL of 10 mg L^−1^ cyanide ions were used to determine the optimal conditions. The optimum ratios of AuNPs to AgNO_3_ for being core-shell structure were studied by using ratio of 0.25 mM AuNPs and 1000 mg L^−1^ AgNO_3_ at 3:1, 3:2 and 3:3 (v/v). In addition, the concentration of AuNPs (0.5, 0.25 and 0.125 mM), AgNO_3_ (500, 800, 1000, 1500 and 2000 mg L^−1^), L-ascorbic acid (300, 500, 1000, 1500 and 2000 mg L^−1^) and NaOH (0.0001, 0.001, 0.01, 0.1 and 1 M) were optimized respectively. Then, the effect of reactions times in range 0–45 min and the pH of samples in the range of 8–14 were evaluated. 

### 2.6. Applications for Real Samples 

The proposed method was applied for the determination of cyanide in the extracted mining wastewater sample and seawater. For first part, the sample was analyzed by Ion chromatography (IC). Moreover, the sample was analyzed with our device to compare with IC results. Prior to analysis, samples were prepared by paper-based head-space extraction for all samples (seawater, drinking water, tap water and wastewater) and the distillation for wastewater.

**Scheme 1:**
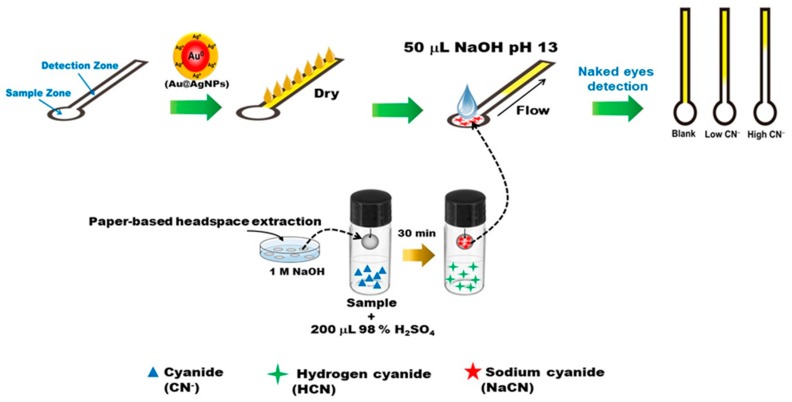
Schematic design of the distance-based paper sensor designs and general analytical methods.

For paper-based headspace extraction, Whatman No.1 filter paper cut to a diameter of 7 mm was immersed in 1 M NaOH solution for 30 min. Then, the paper immobilized with NaOH was hung to the lid of a 25 mL sample vial. Five mL of standard cyanide solutions or samples were added to the sample vial. 200 µL of concentrated H_2_SO_4_ was injected into the vial and closed with the lid for 30 min. Next, the paper was put into sample zone of distance-based paper devices and 50 µL of 0.1 M NaOH was dropped to the sample zone, which carried by capillary action with the flow channel and reacted with Au@Ag NPs through the detection channel as shown in Scheme 1.

For wastewater, the total cyanide was distilled using the SimpleDist^TM^ System [28]. Fifty mL of sample was added into boiling tube and sealed with the screw cap. It was joined into the collection trap which contained 25 mL of 0.25 N NaOH. The vacuum pump was turned on and slowly adjusted valve to provide an air flow bubble rate of 5 bubbles per second for position as viewed in collection trap. Then, 5 mL of 18 M H_2_SO_4_ was slowly added into the boiling tube though the reagent inlet of screw cap and was put in the heating block at 125 °C for 30 min to completely distillation. Finally, the liquid in the collection trap was brought to make the volume up to 50 mL with DI water. The distilled cyanide from wastewater was analyzed using ion chromatography and the distance-based paper device.

## 3. Results and discussion

### 3.1. Characterization of Au@Ag NPs

**Figure 1:**
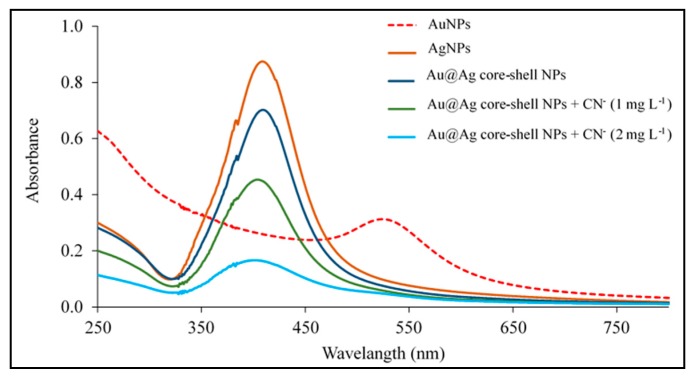
UV-Visible spectra of AuNPs, AgNPs and Au@Ag NPs without and with CN^−^ at 1 and 2 mg L^−1^.

**Figure 2:**
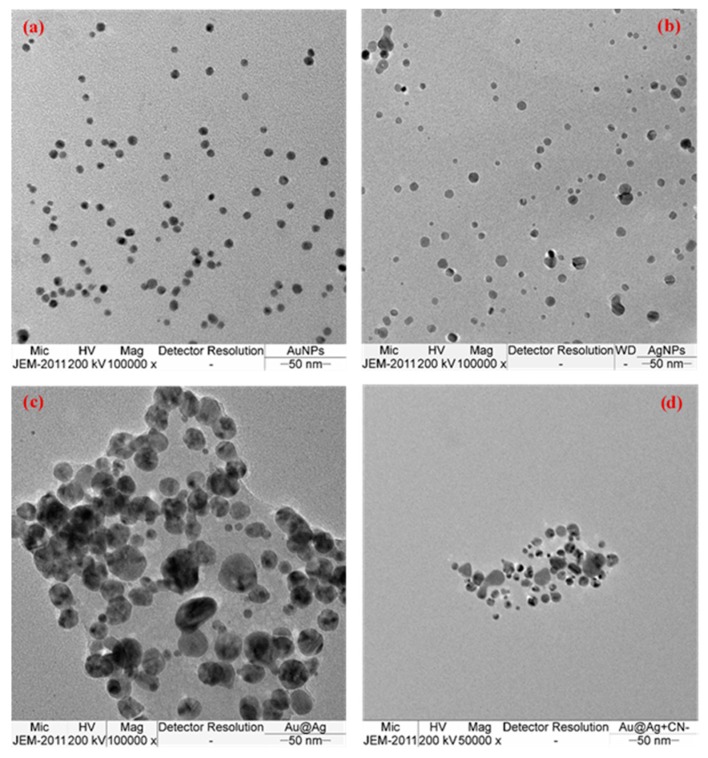
TEM images for (**a**) AuNPs, (**b**) AgNPs, (**c**) Au@Ag NPs and (**d**) Au@Ag NPs in the presence of 20 mg L^−1^ CN^−^.

The Au@Ag NPs were characterized by UV-Visible spectrometer and TEM. The spectra of AuNPs, AgNPs and Au@Ag NPs were shown in Figure 1. AuNPs shows the absorption spectrum at 523 nm whereas the maximum absorption of Au@Ag NPs was found at 409 nm as the same wavelength as AgNPs which can identify the Ag nanoshells on Au@Ag NPs. The absorbance of Au@Ag NPs at 409 nm decreased when the increasing of the cyanide concentration in solution. It indicates that Ag nanoshells of Au@Ag NPs were etched by cyanide ions. From TEM images, the particle size of AuNPs, AgNPs, Au@Ag NPs, and Au@Ag NPs with addition of cyanide were 7.31 ± 1.55, 7.02 ± 2.44, 20.9 ± 7.11, and 9.26 ± 2.77 nm, respectively. The particle size of Au@Ag NPs is larger than AuNPs and TEM image of Au@Ag NPs also exhibited electronic inhomogeneity with a dark core surrounded by a lighter shell (Figure 2c). After adding 50 mg L^−1^ cyanide, the particle size of Au@Ag NPs decreased from 20.9 ± 7.11 to 9.26 ± 2.77 nm (Figure 2d). These results confirms that cyanide can etch Ag nanoshells and an Au core sequentially in the presence of oxygen via Equation (1), leading to the change of dimensional particle, which induces visual color changes from yellow to pink and then to colorless in finally.

(1) $${4\mathrm{Ag}\;+\;8\mathrm{CN}}^{-}{\;+\;2H}_{2}{O\;+\;O}_{2}↔{\;4[\mathrm{Ag}(\mathrm{CN})}_{2}{]}^{-}{\;+\;4\mathrm{OH}}^{-}$$

E^0^ = 0.851 V, Equilibrium constant of this reaction = 9.4 × 10^24^

### 3.2. Study of Reagents Influence on the Length of Au@Ag NPs Color Band on dPADs

The reagent concentration used in the synthesis of bimetallic NPs have a major impact on the shell to core ratio and the thickness of Ag nanoshells have effect on the sensitivity of cyanide detection. Hence, the AuNPs, AgNO_3_, ascorbic acid and NaOH concentration were studied during the preparation of Au@Ag NPs. The concentration of AuNPs was studied at 0.500, 0.250 and 0.125 mM. The colorless length of Au@Ag NPs occurring on the white paper channel after the addition of cyanide was no significant difference among three AuNPs concentrations (Figure S1). However, the highest standard deviation (SD) of the colorless band length on dPADs was found at 0.125 mM AuNPs. To obtain high precision and less reagent consumption, the concentration of 0.250 mM was chosen for the synthesis of Au@Ag NPs in the further experimental. Then, the concentration of AgNO_3_ was investigated in the range of 500–2000 mg L^−1^. The increase of yellow color intensity was observed when AgNO_3_ concentration increased. We supported that the thickness of Ag nanoshells expanded at the high AgNO_3_ concentration. The core/shell NPs with thick silver shells were beneficial for the detection of cyanide on dPADs due to the easy to discriminate the colorless band length by the naked eye, but they suffered from low sensitivity. In contrast, the NPs with thin silver shells were sensitive for the detection of cyanide, but the colorless band length was difficult to resolve by the naked eye (Figure S2). Therefore, the suitable concentration of AgNO_3_ at 1000 mg L^−1^ was selected to compromise the sensitivity and the visual discrimination.

Ascorbic acid was used as the reducing agent for the reduction of Ag^+^ to Ag^0^ during the Au@Ag NPs preparation. The concentration of ascorbic acid was also investigated at 300, 500, 1000, 1500 and 2000 mg L^−1^. The concentration of NaOH was investigated in the concentration of 0.0001, 0.001, 0.01, 0.1 and 1 M. There was no significant difference of the colorless band length but the best visual resolution between yellow color and colorless band length was found at 500 mg L^−1^ ascorbic acid and 1 M NaOH (data not shown). Hence these concentrations were selected as the optimal condition.

### 3.3. Optimization of Detection Conditions on dPADs

**Figure 3:**
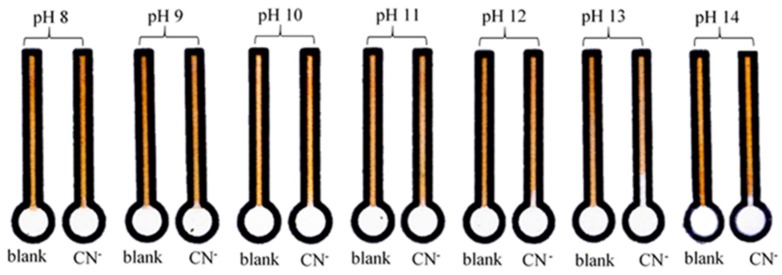
pH effect in the range of 8–14 with the present of CN^−^ 10 mg L^−1^.

The pH effect of the cyanide solution on the sensitivity was studied in the range of pH 8–14. The cyanide solution at pH 13 was chosen because the highest colorless band length was found at this condition (Figure 3). The free cyanide consists of HCN and cyanide ions but 100% of cyanide ions presents in pH > 11. At pH 14, the colorless band distance decreased because the high amount of hydroxide ion can force the backward reaction of equilibrium in Equation (1). 

**Figure 4:**
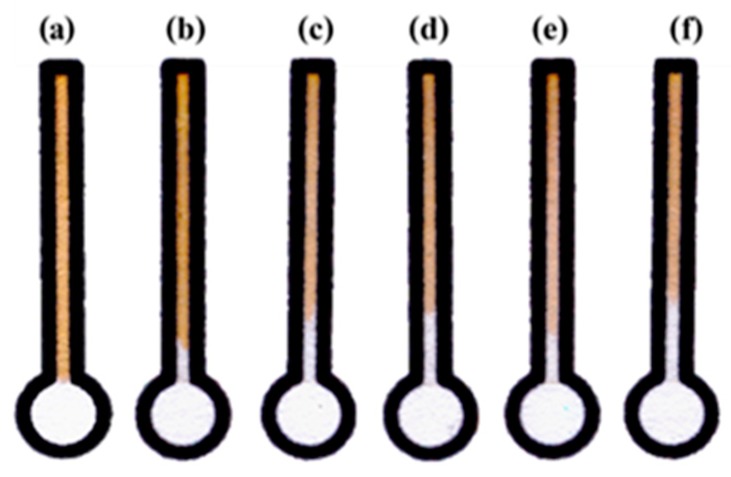
Effect of sample volume at (**a**) 0, (**b**) 25, (**c**) 50, (**d**) 75, (**e**) 100, and (**f**) 50 × 2 µL contained of CN^−^ 10 mg L^−1^.

The influence of sample volume was studied in the range of 25–100 µL. The sample volume at two times of 50 µL gave the highest distance (Figure 4f), but it needs a long analysis time (90 min.). Although, the sample volume increased, the sensitivity of cyanide detection rose up until 75 µL but the analysis time also increased. To obtain the short analysis time, 50 µL of sample volume (Figure 4c) was chosen as the optimum condition for 40 min analysis time (Figure S3).

The stability of devices after storing the prepared distance-based paper device was studied for five weeks. The solutions of Au@Ag core-shell NPs were coated on paper devices and allowed dry before storage at 4 °C. The results were measured by the naked eyes, the intensity has no significant difference from freshly prepared paper devices. The distance signal was decreased at 3.3% after four and five weeks (Figure S4). Thus, this device can be used over than five weeks.

### 3.4. Analytical Performance

**Figure 5:**
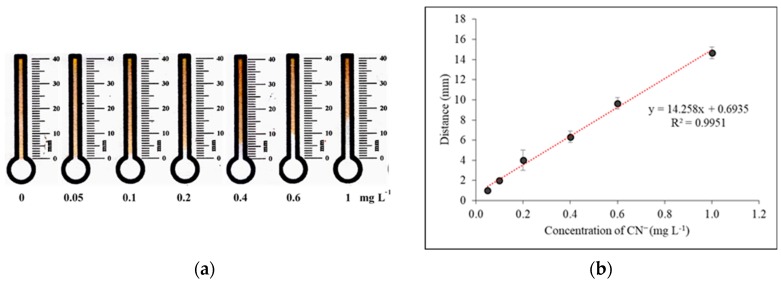
(**a**) Result images of CN^−^ determination in the range of 0.05 – 1 mg L^−1^ with head-space extraction on dPADs and (**b**) Calibration curve for CN^−^ determination in the range of 0.05 – 1 mg L^−1^ with head-space extraction on dPADs.

The calibration curve for the determination of cyanide was shown in Figure S5. The linear range between the distance signal and the concentration were found to be 1–40 mg L^−1^ (R^2^ = 0.9952). The limits of detection (LOD) were obtained at the concentrations as low as 1 mg L^−1^ by naked eyes. The relative standard deviations (%RSD) of three concentrations which were 5, 20, and 40 mg L^−1^ and repeated 10 devices in each concentration were 6.68%, 7.20%, and 5.38%, respectively. Moreover, the sensitivity was increased by paper-based head-space extraction. The enrichment factor was found to be 30-fold and the calibration was found in the range 0.05–1 mg L^−1^ (LOD = 10 μg L^−1^) as shown in Figure 5. The linear range is sufficient for monitoring drinking water where the World Health Organization (WHO) have regulated the maximum level of cyanide in drinking water as 70 µg L^−1^ and the U.S. Environmental Protection Agency (EPA) has established a maximum cyanide contamination level of 200 µg L^−1^ in ambient water. Our lowest naked-eye detectable concentration was 10 μg L^−1^. Although our LOD isn’t lower than those of paper-based sensing platform (0.7 μg L^−1^ and 0.026 ng L^−1^) [15,16], quantification is achieved by measuring color length. Thus, eliminating the need to differentiate hues and intensities by the user and the processing data of each imaging device. 

**Figure 6:**
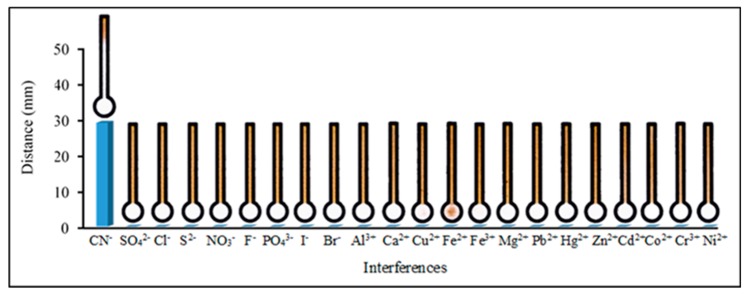
Selectivity of cyanide at 10 mg L^−1^ CN^−^, other anions and cations. The bar shows the white distance on paper-based device for anions and cations detection.

The selectivity of our proposed device was studied at 10 mg L^−1^ cyanide, other anions and cations. As a result, shown in Figure 6, the colorless band length did not present with the addition of other ions, except for cyanide ions. Then, the interferences effect for the determination of cyanide were investigated in the ratio of analyte to interference which in 1:1, 1:10 and 1:100 ratios. It was found that over than or approximate to 100-fold for SO_4_^2−^, Cl^−^, NO^3−^, PO_4_^3−^, S^2−^, F^−^, Br^−^, Ca^2+^, Cu^2+^, Fe^2+^, Fe^3+^, Mg^2+^, Pb^2+^, Zn^2+^, Cd^2+^, Co^2+^, Cr^3+^ and Ni^2+^ have no influences on distance signal of CN^¯^. The tolerated ratio of the other interference effected at 100-fold of I^−^, SCN^−^, and 10-fold of Al^3+^ and Hg^2+^ have an influence on distance signal but it must be emphasized that the I^−^, SCN^−^, Al^3+^ and Hg^2+^ concentration in natural water samples is very low. 

### 3.5. Applications

To evaluate the utility of our proposed devices, dPADs were applied to the determination of cyanide in the extracted mining wastewater samples and real water samples. The result of wastewater was shown in Table 1 which was comparison between the developed method and the IC method. No significant difference between two detection methods were found at the 95% confidence interval by paired *t*-test. The t_calculated_ (0.313 for distillation and 0.273 for headspace extraction) was lower than t_Critical_ (4.303). In addition, paper-based head-space extraction coupled on dPADs was successfully applied for the determination of cyanide in seawater, drinking water, and tap water (Table S1). The recovery of two concentrations for spiked level at 70 and 500 μg L^−1^ in seawater, drinking water, and tap water were found in the range of 93%–108%. Therefore, our device has been successfully applied to determine cyanide in seawater, drinking water, tap water and waste water providing satisfactory precision (% RSD < 7.6) and accuracy (93–108%).

In summary, the distance-based paper device immobilized by Au@Ag NPs was successfully developed for the low cost, rapid, easy to operate, instrument-free, portable determination of cyanide. This analysis is based on the etching reaction of Au@Ag NPs by cyanide ions. The decrease in a yellow color band length of Au@Ag NPs corresponds to the quantity of cyanide; due to a strong change in the color band, the results can be easily detected by naked eye. Paper-based headspace extraction was also coupled with the distance-based paper device to enhance the sensitivity (30-fold enrichment factor). The limit of detection was obtained at the concentrations as low as 10 μg L^−1^ by naked eyes. Our device could be applied to the low-level detection of cyanide in real samples by unskilled personnel and without interference effects.

## Figures and Tables

**Table 1:** Determination of CN^−^ in metallurgical industry wastewater samples.

Samples	Developed Method (±SD, n = 3) Headspace Distillation	IC Method (±SD, n = 3)
	CN^−^ found (mg L^−1^)	CN^−^ found (mg L^−1^)
1	3.96 ± 0.13 3.51 ± 0.17	4.06 ± 0.08
2	9.26 ± 0.14 8.46 ± 0.12	8.58 ± 0.13
3	9.10 ± 0.04 10.58 ± 0.81	9.43 ± 0.15

## Supplementary Materials

The following are available online at https://www.mdpi.com/1424-8220/19/10/2340/s1, Figure S1: The effect of AuNPs for the Au@Ag NPs synthesis with the different concentration of AuNPs at (**a**) 0.500, (**b**) 0.250 and (**c**) 0.125 with the response signal of CN^−^ 10 mg L^−1^. Figure S2: The effect of AgNO_3_ for the Au@Ag NPs synthesis with the different concentration of AgNO_3_ at (**a**) 500, (**b**) 800, (**c**) 1,000, (**d**) 1,500 and (**e**) 2,000 mg L^−1^ on the distance-based paper device with the response signal of CN^−^ 10 mg L^−1^. Figure S3: Detection time for CN^−^ on distance-based device in the range of 0–50 min with 10 mg L^−1^ of CN^−^. Figure S4: Lifetime of CN^−^ on distance-based device. Figure S5: Calibration curve for CN^−^ determination in the range of 1–40 mg L^−1^. Table S1: Determination of CN^−^ in water samples.
