# Supplementary material for: Distance-Based Paper Device Combined with Headspace Extraction for Determination of Cyanide
Source: Sensors (Basel). 2019 May 21;19(10):2340. doi: 10.3390/s19102340 (PMC6566168; doi:10.3390/s19102340)
Supplement: Supplementary file 1 [file sensors-19-02340-s001.pdf]

Supplemental Information

Distance-based paper device combined with headspace extraction for determination of cyanide

Papichaya Khatha[a], Thanyarat phutthaphongloet[a], Phenphitcha timpa[a], Benjawan Ninwong[a], Kamolwich Income[a,b], Nalin Ratnarathorn[a,b] ,Wijitar Dungchai[a,b]

[a]*Organic Synthesis, Electrochemistry & Natural Product Research Unit, Department of Chemistry, Faculty of Science, King Mongkut's University of Technology Thonburi, Prachautid Road, Thungkru, Bangkok, 10140, Thailand.*

[b]*Applied Science & Engineering for Social Solution Unit, Faculty of Science, King Mongkut's University of Technology Thonburi, Prachautid Road, Thungkru, Bangkok, 10140, Thailand.*

Corresponding author: Asst. Prof. Dr. Wijitar Dungchai

E-mail: wijitar.dun@kmutt.ac.th

Fax: +66-2-470-8840

Tel: +66-2-470-9553

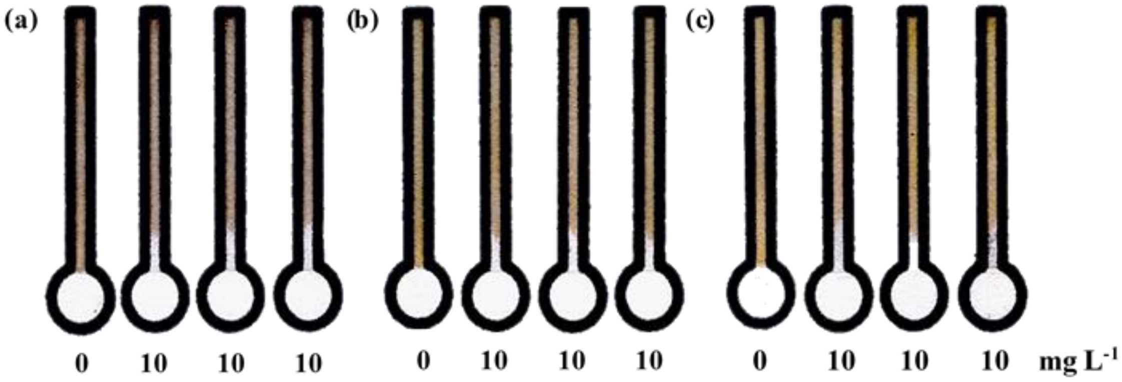

Fig. S1 The effect of AuNPs for the Au@Ag NPs synthesis with the different concentration of AuNPs at (a) 0.500, (b) 0.250 and (c) 0.125 with the response signal of CN^- 10 mg L^{-1}

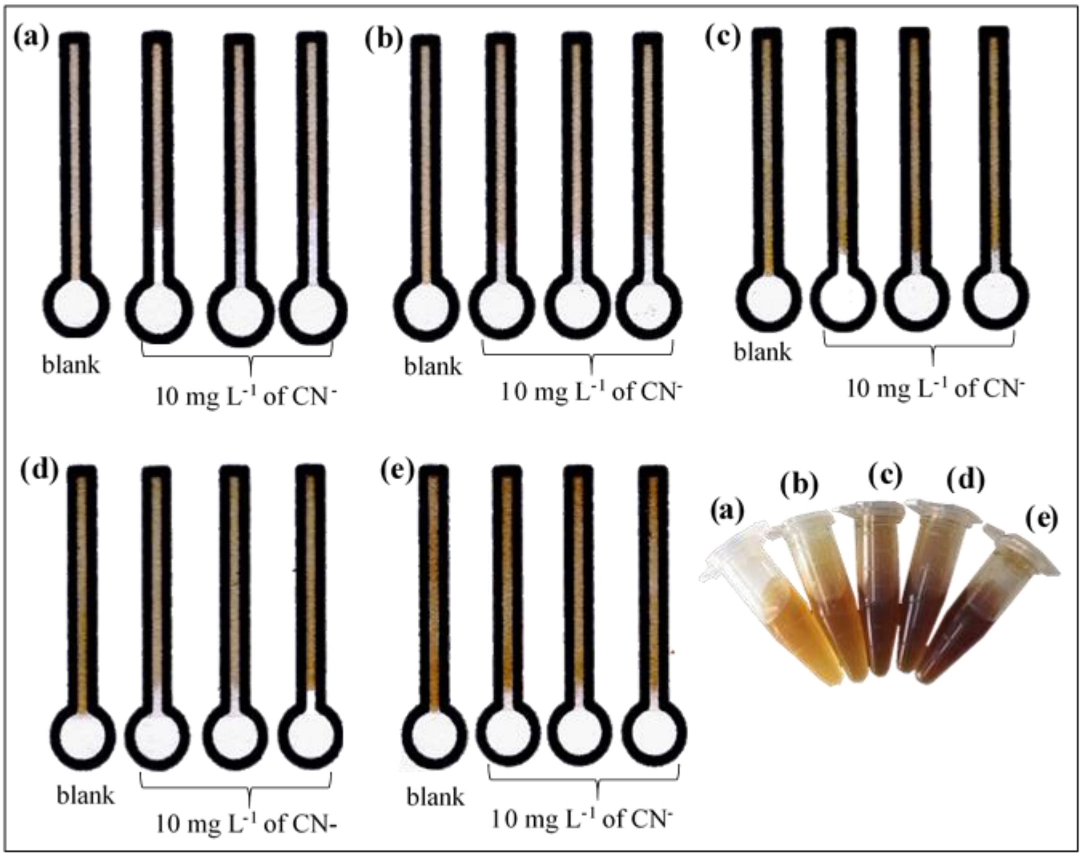

Fig. S2 The effect of $AgNO_3$ for the Au@Ag NPs synthesis with the different concentration of $AgNO_3$ at (a) 500, (b) 800, (c) 1,000, (d) 1,500 and (e) 2,000 mg L^{-1} on the distance-based paper device with the response signal of CN^- 10 mg L^{-1}

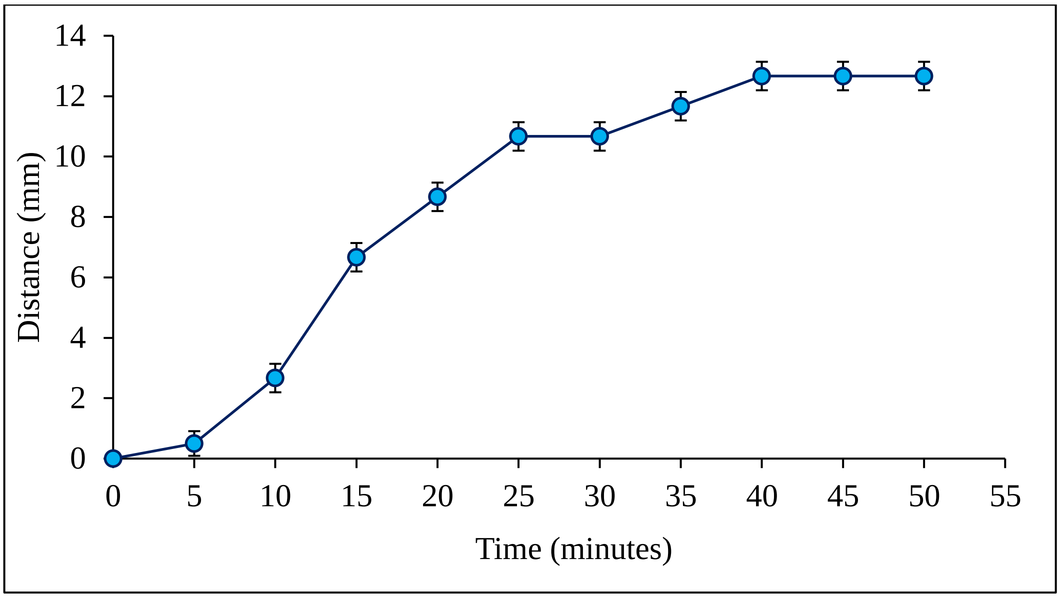

Fig. S3 Detection time for CN^- on distance-based device in the range of 0 – 50 minutes with CN^- 10 mg L^{-1}

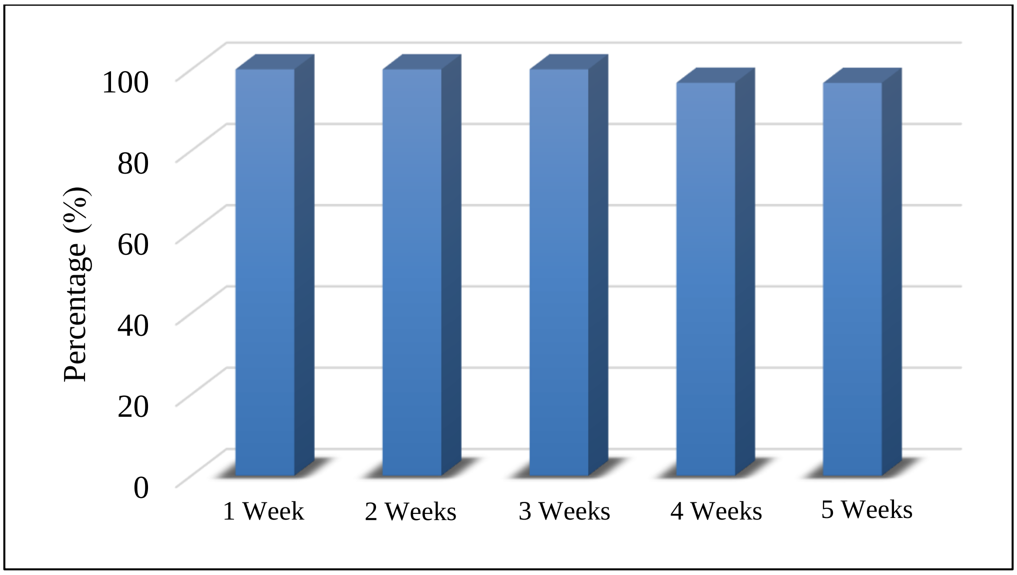

Fig. S4 Lifetime of CN^- on distance-based device

(a)

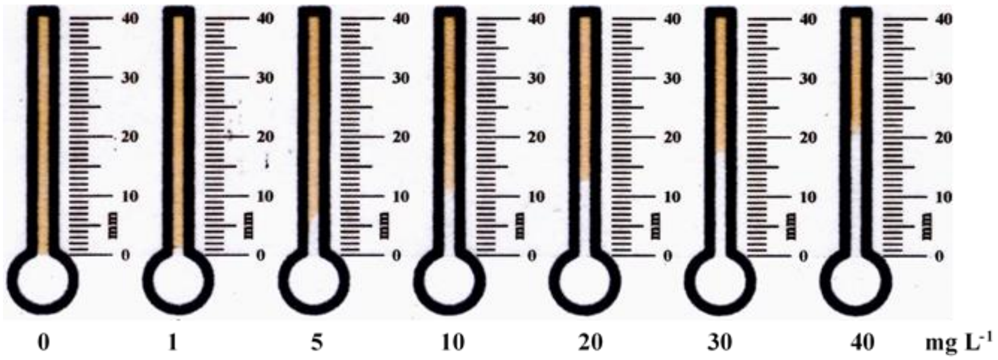

(b)

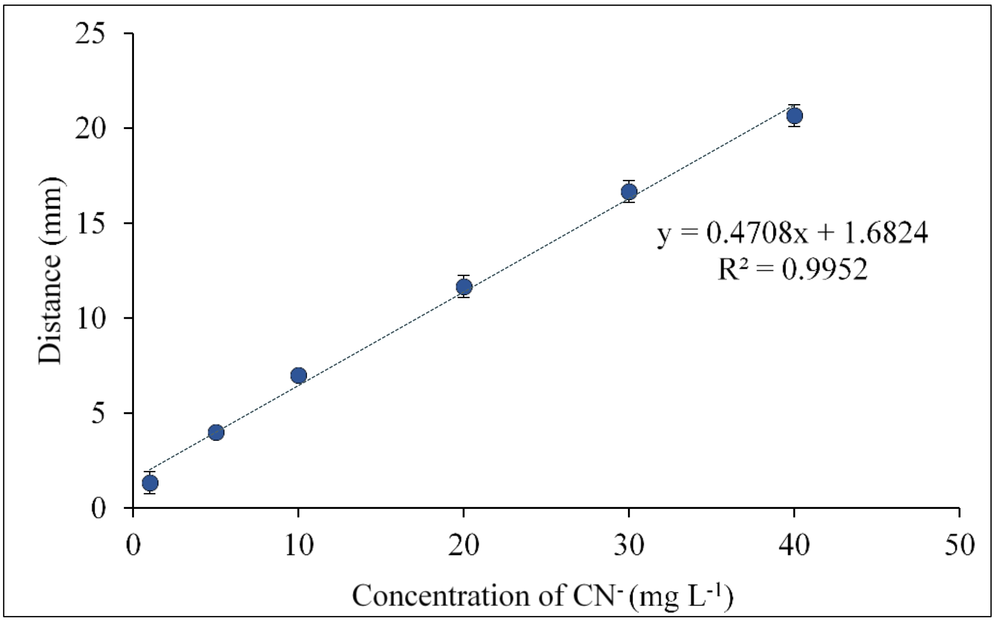

Fig. S5 Calibration curve for CN^- determination in the range of 1 – 40 mg L^{-1}

Table S1 Determination of CN^- in water samples

**Samples**	**Spiked level (μg L^{-1})**	**CN^- found (μg L^{-1})**	**Recovery (%)**
Seawater	0	N.D.	
	70	70 ± 4.0	100
	500	536 ± 40.3	107
Drinking water	0	N.D.	
	70	70 ± 4.0	100
	500	540 ± 11.8	108
Tap water 1	0	N.D.	
	70	68 ± 4.0	97
	500	466 ± 40.2	93
Tap water 2	0	N.D.	
	70	70 ± 4.0	100
	500	470 ± 40.5	94
Tap water 3	0	N.D.	
	70	68 ± 4.0	97
	500	536 ± 40.1	107

N.D. = not detected
